# Receptor-Mediated Targeted Delivery of Surface-ModifiedNanomedicine in Breast Cancer: Recent Update and Challenges

**DOI:** 10.3390/pharmaceutics13122039

**Published:** 2021-11-29

**Authors:** Md. Rizwanullah, Mohammad Zaki Ahmad, Mohammed M. Ghoneim, Sultan Alshehri, Syed Sarim Imam, Shadab Md, Nabil A. Alhakamy, Keerti Jain, Javed Ahmad

**Affiliations:** 1 Department of Pharmaceutics, School of Pharmaceutical Education and Research, Jamia Hamdard, New Delhi 110062, India; mdrizwanullah54@gmail.com; 2 Department of Pharmaceutics, College of Pharmacy, Najran University, Najran 11001, Saudi Arabia; mzahmad@nu.edu.sa; 3 Department of Pharmacy Practice, College of Pharmacy, AlMaarefa University, Ad Diriyah 13713, Saudi Arabia; mghoneim@mcst.edu.sa; 4 Department of Pharmaceutics, College of Pharmacy, King Saud University, Riyadh 11451, Saudi Arabia; salshehri1@ksu.edu.sa (S.A.); simam@ksu.edu.sa (S.S.I.); 5 Department of Pharmaceutics, Faculty of Pharmacy, King Abdulaziz University, Jeddah 21589, Saudi Arabia; shaque@kau.edu.sa (S.M.); nalhakamy@kau.edu.sa (N.A.A.); 6 Department of Pharmaceutics, National Institute of Pharmaceutical Education and Research (NIPER)—Raebareli, Lucknow 226002, India; keertijain.02@niperraebareli.edu.in

**Keywords:** breast cancer, multidrug resistance, nanoparticle, surface-modification, receptor-mediated, targeted delivery

## Abstract

Breast cancer therapeutic intervention continues to be ambiguous owing to the lack of strategies for targeted transport and receptor-mediated uptake of drugs by cancer cells. In addition to this, sporadic tumor microenvironment, prominent restrictions with conventional chemotherapy, and multidrug-resistant mechanisms of breast cancer cells possess a big challenge to even otherwise optimal and efficacious breast cancer treatment strategies. Surface-modified nanomedicines can expedite the cellular uptake and delivery of drug-loaded nanoparticulate constructs through binding with specific receptors overexpressed aberrantly on the tumor cell. The present review elucidates the interesting yet challenging concept of targeted delivery approaches by exploiting different types of nanoparticulate systems with multiple targeting ligands to target overexpressed receptors of breast cancer cells. The therapeutic efficacy of these novel approaches in preclinical models is also comprehensively discussed in this review. It is concluded from critical analysis of related literature that insight into the translational gap between laboratories and clinical settings would provide the possible future directions to plug the loopholes in the process of development of these receptor-targeted nanomedicines for the treatment of breast cancer.

## 1. Introduction

Breast cancer has an extensive history, dating back more than 3500 years to around 1500 B.C. when it was first mentioned by the ancient Egyptians [1]. Breast cancer remains the most commonly diagnosed cancer in women and the leading cause of cancer-related fatalities [2,3]. Breast cancer incidence has increased up to more than 30% in the previous 25 years, despite significant reductions in mortality [3,4]. According to WHO, in 2020, there were 2.3 million women diagnosed with breast cancer and 685,000 deaths globally [5]. Breast cancer had been diagnosed in 7.8 million women in the previous five years until the end of 2020, making it the most prevalent cancer worldwide [5]. Furthermore, as per WHO, breast cancer accounting for 12% of all new annual cancer cases globally and became the most prevalent form of cancer worldwide as of 2021 [6]. The primary risk factor for breast cancer includes gender, genetic factor, hormonal therapy, lifestyle and dietary habits, and growing age [3,6,7].

Breast cancer is a multifaceted disease characterized by a wide range of molecular profiles with characteristics of biological and clinical features [8]. Depending on the molecular characteristics, breast cancer is broadly classified into four types: (i) luminal A (ii) luminal B, (iii) human epidermal growth factor receptor 2 (HER2)-positive, (with overexpressed HER2 and negative estragon and progesterone receptors), and (iv) basal-like breast cancer (also known as hormone and HER2 negative or triple-negative breast cancer (TNBC)) [3,9]. Treatment options for breast cancer now include surgery (mastectomy and lumpectomy), radiation, chemotherapy, and hormone therapy. Chemotherapeutic options for breast cancer include tamoxifen (estrogen antagonist) for luminal A and luminal B, trastuzumab (antibodies) but chemotherapeutic options for TNBC are limited [3,7].

In this review, notable challenges in breast cancer therapeutic intervention are emphasized. A comprehensive account of surface-modified nanomedicines is discussed that improve the efficacy of loaded therapeutics for breast cancer treatment. The key challenges that need to be addressed to improve the utility of receptor-targeted nanomedicines in clinical settings are also discussed.

## 2. Challenges in Breast Cancer Therapy

Despite considerable advancements in breast cancer treatment, there are still numerous hurdles in accomplishing optimal therapeutic response. Chemotherapy is the primary therapeutic option, either alone or combined with other therapeutic approaches such as radiation or surgery [10,11,12,13]. Lack of specificity and multidrug resistance (MDR) is the significant shortcomings associated with cancer therapeutic intervention [14,15,16,17]. Furthermore, patients receiving chemotherapy should be able to tolerate frequent dosing, which leads to abridged quality of life [18]. The commonly used chemotherapeutic drug regime has several disadvantages, including inappropriate biodistribution, unfocused targeting, suboptimal bioavailability, high toxicity, and potential adverse effects [18]. The development of anticancer MDR in breast cancer could be attributed to ABC transporters, overexpression of the P-glycoprotein (P-gp), MDR associated protein (MRP1), breast cancer resistance protein (BCRP), microtubules alteration, altered enzymes, p-53 tumor suppressor gene are the other significant challenges in breast cancer therapy [12,19,20].

### Multidrug Resistance (MDR) in Breast Cancer

**Figure 1:**
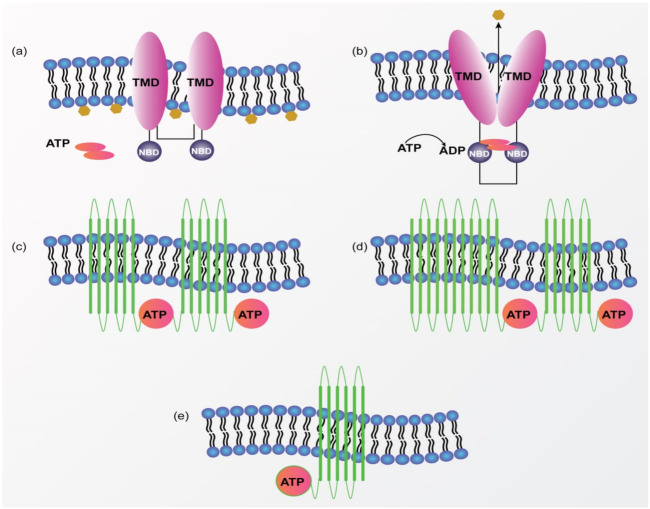
Illustrate the common structure of the ABC transporters and their subfamily. (**a**) ABC transporter with 2 sets of transmembrane domains and 2 nucleotide-binding domains. Orange color structure in the inner membrane indicates substrate molecules. (**b**) The binding of ATP caused the joining of NBD which ultimately leads to a conformational change and transfer of the substrate molecule out of the membrane. (**c**) Common structure of P-glycoprotein (P-gp) as one of the ABC transporters contains 12 TMD and 2 sites for ATP binding. (**d**) Another ABC transporter as Multidrug Resistance Protein 1 (MRP1) also contains 12 TMD and 2 sites for ATP binding similar to the P-gp transport system but also contains an extra 5 TMD at the amino-terminal end. (**e**) Another ABC transporter as Breast Cancer Resistance Protein (BCRP) contains 6 TMD and only 1 site for ATP binding at the amino-terminal end of the TMD. TMD: Transmembrane domain; NBD: Nucleotide-binding domains.

MDR is a complicated mechanism that includes aberrant vasculature, localized hypoxia, a low pH environment, aerobic glycolysis, enzymatic degradation, up-regulated ABC-transporters, an enhanced apoptotic threshold, and increased interstitial fluid pressure, among other conditions that limit the therapeutic action of drug [19,20]. The exact mechanism of MDR in the breast cancer cell is still unclear, it may include enhanced drug efflux, gene mutation, gene amplification or epigenetic alteration, and enhanced xenobiotic metabolism [21]. Additionally, MDR can be caused by deficient apoptotic pathways as a result of malignant transformation, a change in the apoptotic pathway during chemotherapeutic exposure, or alterations in the cell cycle processes that activate checkpoints and impede apoptosis. [20]. To overcome MDR, it is of vital importance to have a thorough understanding of the underlying molecular mechanisms of MDR to develop effective therapeutic options. MDR research showed that drug transport is a highly regulated process, which was later discovered to be regulated by members of the ATP binding cassette (ABC) transporter family of proteins [22]. ABC-transporters are the transmembrane transporter proteins found in cancer cells and are responsible for MDR to anticancer treatment. They act as a membrane-bound molecular pump and utilize the energy from the hydrolysis of ATP to transport various substrates across the biological membrane [20,23] (Figure 1).

**Figure 2:**
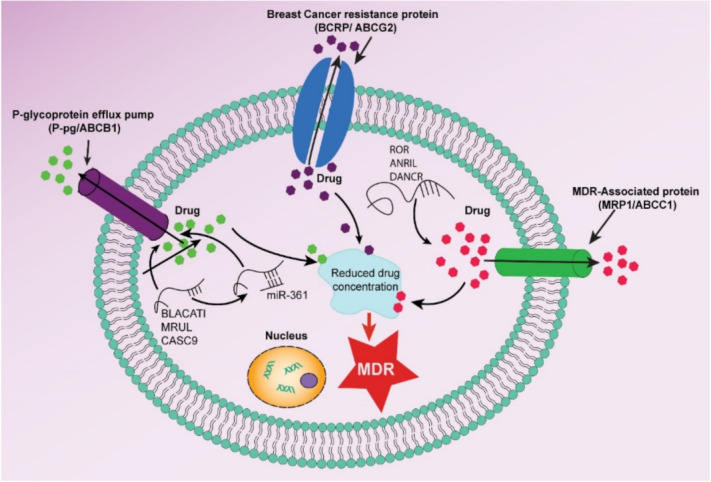
Different ABC transporter overexpressed in breast cancer cells responsible for MDR and failure of chemotherapy.

Among various ABC transporters, the main ABC transporters primarily linked to MDR in breast cancer are P-glycoprotein (P-gp/MDR1/ABCB1), multidrug resistance-associated protein 1 (MRP1/ABCC1), and breast cancer resistance protein (BCRP/ABCG2) [20,22,24]. Based on sequence analogy and structural organization, the ABC transporter family has been subdivided into seven subfamilies (ABCA-ABCG) [20,24]. So far, there are 49 different ABC transporter have been identified in humans, however, bacteria and parasites contain more [22,25]. The overexpression of particular ABC transporter on breast cancer cells results in MDR, which is the prominent contributing factor to chemotherapy failure (Figure 2). P-gp/ABCB1 was the first ABC transporter discovered by Juliano and Ling in 1976 and was identified as overexpressed in breast cancer cells and responsible for MDR [26]. P-gp is a multidrug efflux pump with a broad substrate specificity with 12 transmembrane domains and two ATP-binding sites [20,22,24,25]. It is involved in the elimination of neutral and cationic hydrophobic substances from cells (etoposide, vincristine, vinblastine, daunorubicin, doxorubicin, and paclitaxel) [20]. MRP1 is the first member of the C subfamily and act as a lipophilic anion efflux transporter with a broad substrate specificity that is expressed in a wide variety of organ and cell including breast cancer. It acts as a lipophilic anion efflux pump and overexpression of MRP1 leads to cells becoming resistant to a wide range of anticancer drugs, including doxorubicin [20,22].

BCRP was first isolated from MDR human breast cancer cells [27]. It is mostly located in the plasma membrane of the placenta, small intestine, prostate, brain, liver, and ovaries, and it is overexpressed in a wide range of cancer including breast tumors [20,22]. BCRP, as an efflux transporter, pump a wide range of xenobiotic and toxic chemicals, including anticancer drugs, and is associated with MDR to anticancer therapeutics such as methotrexate (MTX), camptothecin, anthracyclines, flavopiridol, and antifolates [20,22].

As discussed earlier, the clinical subtypes of breast cancer have different therapeutic approaches. However, the molecular pathway revealed that this disease shares several common characteristics, specifically, disruption to the phosphatidylinositide 3-kinase (PI3K)/Akt pathway, miRNA levels, and epigenetic gene transcription modulation [28]. The PI3K/Akt signaling pathway plays a significant role in the regulation of numerous physiological responses, including cell growth and survival in both normal and malignant breast tissue [28]. It serves as a site of confluence for all clinical subtypes of breast cancer, and anomalies in this pathway occur in 70% of breast cancers regardless of subtype [29]. Resistance to tamoxifen and trastuzumab is influenced by changes in this pathway, which serves as a convergence between HER2 signaling and ER-regulated gene transcription [28,30]. Furthermore, it has been associated with P-gp overexpression and results MDR [28,30]. From the last few decades, it has been demonstrated that aberrations to the expression of microRNA (miRNA) influence the cancer prognosis and consequences [28,31]. miRNAs are short, noncoding RNAs (about 22 nucleotides) that attach to mRNA, preventing translation and speeding up mRNA deadenylation and degradation, resulting in gene silencing effect. A number of miRNAs have been linked to MDR in breast cancer [28,32]. The term epigenetics refers to the study of persistent changes in gene expression potential that occur during development and cell proliferation [33]. Several epigenetic changes occur during carcinogenesis, including genome-wide loss of DNA methylation, localized hypermethylation, universal changes in histone modification, and modifications in the expression of miRNAs [21]. Accordingly, breast cancer cells develop a DNA methylation mechanism to silence tumor repressor genes [34]. This DNA methylation is involved in the reprogramming of cells during drug resistance phenotype transformation [34]. Specific DNA methylation markers have been reported in about 30% of MDR breast cancer cells. [35]. Thus, it can be concluded that the status of DNA methylation plays a critical role in mediating the MDR in breast cancer.

## 3. Nanotechnology-Mediated Drug Delivery in Breast Cancer

**Figure 3:**
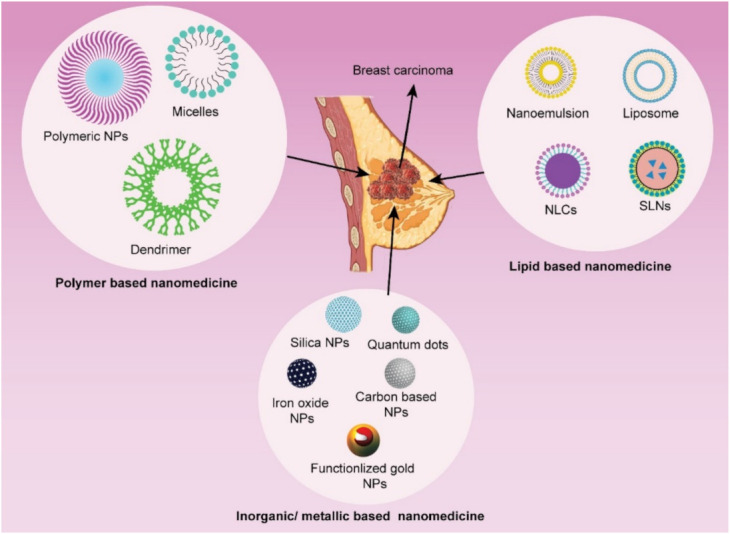
Different types of nanoparticulate systems utilized in receptor-mediated targeted drug delivery in breast cancer.

Most of the cancer chemotherapeutics are typically hydrophobic, nontargeted, and toxic in nature that can have serious adverse effects [36]. Over the last three decades, anthracyclines and taxanes have been two cornerstone chemotherapeutics utilized in advanced breast cancer chemotherapy [37]. However, cardiotoxicity has been linked to the anthracycline family, particularly doxorubicin formulations [38]. Similarly, taxanes have been reported to induce bone marrow depression, hypersensitivity reactions, and dose-dependent neurotoxicity [39,40,41]. In recent decades, advancement in the therapeutic approach of breast cancer has been significantly improved by the surface-modified nanomedicine. Nanotechnology provides a potential approaches alternative to conventional chemotherapeutics, conquering MDR by encapsulation or conjugating therapeutic moieties to nanometric carriers [10,11,12,13,16,18]. The emerging nanomedicine provides desirable drug delivery features, including solubilized lipophilic therapeutic agents, improved biocompatibility, diminished drug degradation in blood, reduced drug clearance, enhanced passive or active targeting, significantly decreased nonspecific cellular internalization, and negligible side effects [15,16,42,43]. Additionally, drug-loaded nanocarriers have recently proved improved treatment efficacy against MDR breast cancer and reduced detrimental side effects, when compared to traditional, nontargeted therapeutic drugs either single or in combination. Furthermore, surface-modified nanomedicine also has the potential to successfully target and remove breast cancer stem cells, which may play a significant role in breast cancer instigation, recurrence, and chemo/radiotherapy resistance [44]. Hence, rapidly evolving nanotechnology has the ability to address the intricate and multifaceted mechanisms of MDR, allowing nanomedicine to offer a novel and promising approach to combat and conquer chemoresistance against breast cancer. With the rapid growth of nanotechnology over the last 30 years, numerous nanomaterials have been developed, but only a few nanocarrier-based systems are being used in nanomedicine, and even fewer can fulfill the approval criteria of the US FDA [15]. The nanocarrier system can be broadly classified as lipid-based, polymeric, inorganic, and a hybrid of lipid and polymeric nanoparticles (Figure 3) [10,11,12,13,15,16,18].

The lipid-based nanocarriers have been proved to be superior over polymeric and inorganic nanocarriers in terms of biocompatibility, safety, and biodegradability. Lipid nanocarrier drug delivery system can be sub-categorized into nanoemulsion, self-nano emulsifying drug delivery system (SNEDDS), liposomes, solid lipid nanoparticles (SLN), and nanostructured lipid carrier (NLC). In SNEDDS and nanoemulsion (oil-in-water), the modifying agent such as probes or imaging agents and lipophilic anticancer drugs are enclosed in oil droplets for specific tumor site delivery. In liposomes, the imaging/diagnostic agent can be enclosed with anticancer therapeutics either in an aqueous core or lipid bilayer according to their physicochemical characteristics (hydrophilicity/lipophilicity). Solid matrix lipid nanoparticles such as SLN (composed of solid lipid only) and NLC (composed of both solid and liquid lipid) entrapped the lipophilic therapeutics and imaging agents into the lipid phase of this delivery system. The various attributes of liposomes (like unique structure, and great flexibility to accommodate both hydrophilic and lipophilic therapeutic/imaging agents) make it promising therapeutic/imaging compared to other lipidic nanocarrier systems. However, optimizing lipid nanomedicines with desirable size distribution, shape, surface charge, and stability is quite challenging in itself. Recently, exploring cancer cell-specific drug delivery to improve the antitumor efficacy of lipid-based nanocarriers by surface functionalization with various ligands and peptides is contemplated in extensive research work. These lipid nanocarriers are now being designed to encapsulate or conjugate multifunctional components such as anticancer agents, targeting moieties, antibodies, etc. in one single formulation, therefore can be called complex nanoproducts [10,11,12,13,15,16,18]. Inorganic nanoparticles have also been widely investigated for the delivery of anticancer therapeutics and have demonstrated encouraging findings in pre-clinical studies that prompt for their translation clinical trials. Gold nanoparticles, quantum dots, superparamagnetic iron oxides are examples of inorganic nanoparticles. Whereas carbon nanotubes, polymeric micelles, dendrimers, nanoemulgel, mesoporous silica nanoparticles, metallic nanoparticles, and polymer-drug conjugated are the potential polymeric or organic nanocarriers. Surface modification of these lipid-based, organic and inorganic nanoparticles with targeting moieties, ligands, or other compounds is possible, providing these nanocarriers multifunctional properties [10,11,12,13,15,16,18,19,43,45].

Lipid-polymer hybrid is a promising new generation nanocarrier system and concept has originated from the fusion of liposomes and polymeric nanoparticles. It has polymeric core enclosed by a similar kind of lipid bilayer shell. Many researchers are exploring its potential as drug delivery nanocarrier system particularly for anticancer therapeutics in current scenario [44,45].

Among the plethora of potential nanocarriers, liposomes are the most widely explored nanocarrier and have also been established as an anticancer nanomedicine in the form of Doxil^®^. It has been used against Kaposi’s sarcoma, refractory breast and ovarian cancer with a circulating half-life of 74 h in breast cancer patients, compared to approximately 5 min t_1/2_ for free drug [46]. The prolonged circulation half-life of Doxil^®^ is attributed to pegylation (coating of liposome surface with polyethylene glycol) which guards the liposomes against recognition by the mononuclear phagocyte system and offers a stabilization effect by mitigating adherence to cells, blood vessel walls, and other surfaces. Furthermore, it was also observed that the risk of cardiotoxicity associated with a peak concentration of drug was significantly reduced with Doxil^®^ in breast cancer patients in comparison to cancer patients who developed cardiotoxicity when treated with free drug [47]. Liposomes, as nanocarriers, can be tailored the way they can offer passive or active targeting, and thereby overcome MDR and reduce side effects of drugs. The regulation of P-gp by liposomes is another key strategy for improving anticancer medication and therapeutic efficacy in MDR cancer cells [48]. Polymeric micelles represent another kind of potential nanocarriers system with promising delivery of chemotherapeutic agents to both nonresistant and MDR cancer cells [49]. A polymeric micelle is formed primarily when the hydrophobic section of a block copolymer is forced into the aqueous phase, while the hydrophilic portion faces outward to form a shell. Nanoemulsions are another kind of nanocarriers that are being explored as an anticancer drug delivery system in MDR [50]. Most of the nanoemulsions are oil in water types, in which oil droplets are dispersed in the continuous phase (water) at the size of about 10–100 nm. Because of their great solubilization capacity for lipophilic drugs in their oily phase, the oil-in-water type is the most widely used emulsion system. Among nanoparticulate systems, polymer nanoparticles, solid lipid nanoparticles, inorganic nanoparticles, polymeric conjugates, carbon nanotubes, and dendrimers have all been extensively investigated for their potential to overcome MDR in breast cancer.

## 4. Surface-Modified Multifunctional Nanomedicine for Breast Cancer

**Figure 4:**
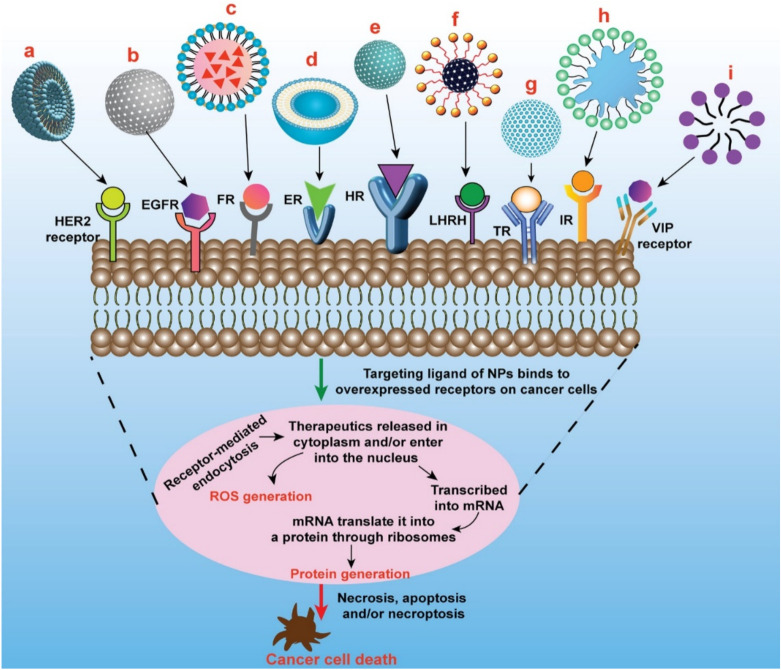
Schematic presentation of different types of targeted nanomedicine mediated through overexpressed receptors in breast cancer to induce cancer cell death/apoptosis. **a.** Oligoclonal antibody conjugated liposomes loaded with doxorubicin induce significant cell killing in HER2-overexpressed BT-474 breast cancer cells. **b.** GE11 peptides conjugated PEGylated PLGA nanoparticles loaded with curcumin induce significant cell killing in EGFR-overexpressed MCF-7 breast cancer cells **c.** Folate conjugated nanostructured lipid carriers loaded with curcumin induce significant inhibition of tumor growth compared to nontargeted nanomedicine in MCF-7 xenograft mice model. **d.** Doxorubicin-loaded liposomes surface-grafted with tamoxifen (ER antagonist) cause increased cellular/nuclear uptake of loaded therapeutics and induce more cell death compared to plain liposomes in ER overexpressed MCF-7 cells. **e.** PLGA/HA copolymers nanoparticles loaded with docetaxel causes increased cellular uptake and cytotoxicity in MDA-MB-231 breast cancer cells through CD44-mediated endocytosis. **f.** LHRH-conjugated PEGylated magnetite nanoparticles exhibited enhanced uptake in triple-negative breast cancer cells. **g.** Transferrin-capped mesoporous silica nanoparticles loaded with doxorubicin cause increased internalization in MCF-7 cells through transferrin-mediated endocytosis. **h.** Doxorubicin-loaded leukocyte mimicking nanoformulation (leukosomes) exhibited enhanced accumulation in tumor and significantly reduces the tumor volume. **i.** VIP functionalized phospholipid micelles loaded with pararubicin cause increased anticancer activity in MCF- 7 cells. HER2—Human Epidermal Growth Factor Receptor2; EGFR—Epidermal Growth Factor Receptor (HER1); FR—Folate Receptor; ER—Estrogen Receptor; HR—CD44/Hyaluronan Receptor; LHRH—Luteinizing Hormone-Releasing Hormone Receptor; IR—Integrin Receptor; VIP—Vasoactive Intestinal Peptide Receptor.

The current focus in pharmaceutical development and innovation is shifting towards the ‘smart drug’ paradigm, in which improved efficacy and minimal toxicity are of prime significance. This could be attained through delivering the cancer chemotherapeutics by targeting tumor-specific receptors. Identification of particular surface receptors in cancer cells that allows targeted delivery of cancer chemotherapeutics or other therapeutic agents like small interfering RNA (siRNA), can remarkably minimize the undesirable effects and upsurge the efficacy of cancer therapy [51,52]. Many breast cancer cell markers act as potential targets for the precise delivery of cancer chemotherapeutics which include overexpressed estrogen receptor (ER), progesterone receptor, HER2 receptor, folate receptor (FR), epidermal growth factor receptor (EGFR), transferrin receptor (TfR), integrin receptor, nucleolin receptor, CD44 receptor, etc. on the surface of breast cancer cells [53,54]. These receptors are highly overexpressed on the breast cancer cells as compared to normal cells and serve as a captivating way to deliver many potent anticancer agents loaded nanoparticulate system [55,56]. Various receptor-specific therapeutics utilized in breast cancer treatment are presented graphically in Figure 4. The major advantages of receptor-mediated active targeting in breast cancer are (i) it can prevent or at least substantially limit untoward effects of the anticancer agent on healthy tissues; (ii) it can enhance internalization loaded therapeutics by cancer cells; and (iii) it can overcome (at least in part) resistance mechanisms that are based on the active efflux of loaded therapeutics from cancer cells [57].

Although receptor-targeted nanomedicine is being explored for a long time, yet it is challenging to find new ideal ligands that could fulfill the pre-requisites of the targeted drug delivery. For receptor-targeted nanomedicine, the ligand should exhibit specificity as well as a high binding affinity towards its receptor. The density of ligands on the nanocarriers is important for optimal interaction with the receptors. If the density of the ligand is too high, the binding efficiency may reduce owing to steric hindrance. The ligand should have appropriate functional groups that can be modified chemically for conjugation onto the drug-loaded nanocarriers without affecting its receptor binding affinity. The receptor should facilitate the internalization of nanocarriers for intracellular delivery of active cytotoxic agents. These intricate requirements are the main challenges in taking receptor-targeted nanomedicine to the clinical stage [58,59].

### 4.1. Targeting of ErbB Receptor

Breast tumors express elevated levels of growth factors, and their receptors and breast cancer cells show either autocrine or paracrine-stimulated growth [60]. The ErbB tyrosine kinase receptors (type I tyrosine kinases receptors) are widely investigated growth factor receptor systems in breast cancer. This receptor system consists of four homolog receptors: ErbB1 (HER1/EGFR), ErbB2 (HER2/neu), ErbB3 (HER3), and ErbB4 (HER4) [61,62,63]. EGFR receptor is overexpressed in 15–20% of breast cancer cells and HER2 receptor is overexpressed in about 20–25% of breast cancer cells [64]. The overexpressed ErbB receptors show aggressive clinical behavior on breast carcinoma. Due to these reasons, treatments focusing on these receptors have the potential to be valuable anticancer therapies [65].

Farasat and coworkers developed oligoclonal antibody conjugated DOX-loaded liposomes and compared its efficiency with nonconjugated liposomes in targeting HER2-overexpressing and HER-2 negative breast cancer cell lines. In the results, scientists observed that oligoclonal antibody conjugated DOX-loaded liposomes showed an increased binding efficiency to HER2 overexpressing BT-474 and SK-BR-3 breast tumor cells and HER2-negative human breast epithelial MCF-10A cell lines. The results showed receptor-specific binding of targeted liposomes to SK-BR-3 and BT474 cells. This could be due to liposomes conjugated with oligoclonal antibodies compared to monoclonal antibodies-conjugated liposomes. However, oligoclonal antibody conjugated liposomes exhibited higher cytotoxicity in HER2-positive tumor cells compared to the nontargeted liposomes [66]. In another study, Duan et al. developed Fab’ (antigen-binding fragments cut from trastuzumab) and trastuzumab modified curcumin-loaded PEG -PLGA NPs (Fab′-Cur-NPs and TMAB-Cur-NPs). The in vitro results showed a prominent ability to kill HER2-overexpressing BT-474 breast cancer cells and significant accumulation compared to the untargeted NPs. However, there was no significant difference in the accumulation in HER2 negative MDA-MB-231 cells. A pharmacokinetic study in Sprague-Dawley rats showed an enhancement in the half-life (t_1/2_) and absolute bioavailability of Fab′-Cur-NPs by 5.30 and 1.76 folds respectively as compared to that of TMAB-Cur-NPs. Moreover, the biodistribution study showed that Fab′-Cur-NPs demonstrated significantly higher tumor accumulation compared to TMAB-Cur-NPs [67].

In a research investigation, EGFR-targeting GE11 peptides conjugated curcumin-loaded PLGA-PEG NPs demonstrated enhanced delivery of curcumin to EGFR-expressing MCF-7 cells compared to free curcumin and non-EGFR targeted NPs. Moreover, treatment of breast cancer cells and tumor-bearing mice with these EGFR targeted curcumin NPs led to decreased phosphoinositide 3-kinase signaling, a significant reduction in cancer cell viability, low drug clearance from the circulation, and significant suppression in tumor burden as compared with free curcumin and nontargeted NPs [68]. Herceptin-conjugated PLA-D-α-tocopheryl polyethylene glycol 1000 succinate (TPGS) blend NPs showed significantly higher cellular uptake in SK-BR-3 cells. In vitro cell line investigations demonstrated that targeted NPs had a 9.6-fold lower IC_50_ value in comparison with untargeted NPs [69]. Similarly, Kutty and Feng, developed cetuximab-conjugated TPGS micelles for targeted delivery of docetaxel to TNBC cells. In vitro investigations revealed cetuximab-conjugated TPGS micelles exhibited remarkably higher cellular uptake as compared to unconjugated NPs on MDA-MB-468 and MDA-MB-231 breast cancer cells which overexpressed EGFR on the cell surface. In vitro cytotoxicity study showed that cetuximab-conjugated TPGS micelles exhibited 205.6 and 223.8-folds higher efficiency compared to free drug solution against the MDA-MB-468 and MDA-MB-231 cell lines respectively [70].

Milane et al. synthesized an EGFR-targeted paclitaxel/lonidamine co-loaded PLGA/PEG/EGFR targeting peptide combined poly(ε-caprolactone) NPs for the treatment of MDR breast tumor cells. The peptide delivery system actively targeted MDR cells by exploiting the EGFR expression. The targeting system inhibited the Warburg effect and promoted mitochondrial binding of pro-apoptotic Bcl-2 proteins (by lonidamine), while hyper stabilizing the microtubules (by paclitaxel). This strategy enhanced the therapeutic index of both drugs and showed the potentiating effect of combination therapy in the treatment of MDR breast cancer [71]. In another study, Dilnawaz et al. developed HER2 antibody conjugated paclitaxel and/or rapamycin-loaded glycerol monooleate coated magnetic NPs which exhibited an enhanced uptake in the MCF-7 human breast cancer cell line. In vitro cytotoxicity in the same cell line showed that the targeted paclitaxel-loaded magnetic NPs exhibited ~24-fold reduction in IC_50_ value than native paclitaxel and ~3 folds lower IC_50_ value than untargeted paclitaxel-loaded magnetic NPs. In the case of rapamycin, the targeted magnetic NPs exhibited ~71-fold reduction in IC_50_ value than native drug and ~10 folds lower IC_50_ value than untargeted magnetic NPs. In the case of combined drug formulation, the targeted NPs exhibited ~55-fold reduction in IC_50_ value than native drugs and ~7-folds lower IC_50_ value than untargeted NPs. Therefore, antibody-doped NPs could be used as a promising drug carrier for the delivery of active therapeutic agents in targeted breast cancer therapy [72].

Acharya et al. developed EGFR antibody conjugated rapamycin-loaded polymeric PLGA NPs which showed that EGFR conjugated immunonanoparticles exhibited nearly 13-folds higher cellular uptake and antiproliferative activity than unconjugated NPs in MCF-7 cell line [73]. In another study, Sun et al. demonstrated that coumarin 6-loaded PLGA/montmorillonite-trastuzumab NPs exhibited significantly higher cellular uptake efficiency than the nontargeted NPs. The outcome of in vitro cytotoxicity investigation on SK-BR-3 cells further proved the targeting effects of the antibody conjugation. The therapeutic effect of the HER2 decorated NPs was 12.74-fold and 13.11-fold greater than that of the untargeted NPs and higher than taxols in terms of IC_50_ value, respectively [74].

These studies conclude that nanotechnology-based targeting of antineoplastic agents against ErbB receptors can be further explored to enhance their therapeutic efficacy against various breast cancers.

### 4.2. Targeting of Folate Receptor

Folic acid (vitamin B9) is water-soluble, low-molecular-weight (441.4 g/mol) compound that is essential in normal mitotic cell division and growth. Folic acid is very significant in infancy and pregnancy; however, it could also participate in nourishing some of the cancers [75]. Folic acid (FA) or folate has a very high affinity towards FR and FRs (especially the α-isoform) are expressed in a relatively higher percentage in breast cancer cells therefore these receptors can be successfully implicated in the fabrication of FR-mediated targeted drug delivery systems [76,77]. Folate conjugation has been largely exploited for receptor-targeted therapy of breast cancer [78]. FRs are cell surface glycosyl phosphatidylinositol anchored proteins that not only bind FA but also 5-methyltetrahydrofolate [79,80]. The FRs present on the cell surface binds the folate ligand and then the complexes are internalized into the cell through receptor-mediated endocytosis. Once internalized, these complexes release folate intracellularly in the endosomes, and the discharged FRs recycle back to the cell surface [81,82].

Folate-conjugated amphiphilic cyclodextrin paclitaxel NPs developed by scientists showed significantly higher cellular uptake and cytotoxicity in 4T1 breast cancer cells compared to untargeted NPs. Paclitaxel-loaded folate decorated NPs significantly reduced tumor burden and survival time compared to untargeted NPs [83]. Thapa et al. developed FA-decorated cisplatin, and docetaxel co-loaded liquid crystalline NPs, which exhibited significantly higher cellular uptake by FR-overexpressing MDA-MB-231 cells to a greater extent than FR-negative A549 cells in comparison to nontargeted NPs, attributed to folate receptor-mediated endocytosis of the targeted NPs. The increased expression of various apoptotic markers including Bax, p21, and cleaved caspase-3 along with improved antimigration effects in MDA-MB-231 breast cancer cells following treatment demonstrated that the folate decorated NPs can be used for successful treatment of metastatic breast cancer. Moreover, folate decorated NPs exhibited significantly higher anticancer efficacy both in vitro as well as in the MDA-MB-231 tumor xenograft model compared to nontargeted NPs [84].

**Figure 5:**
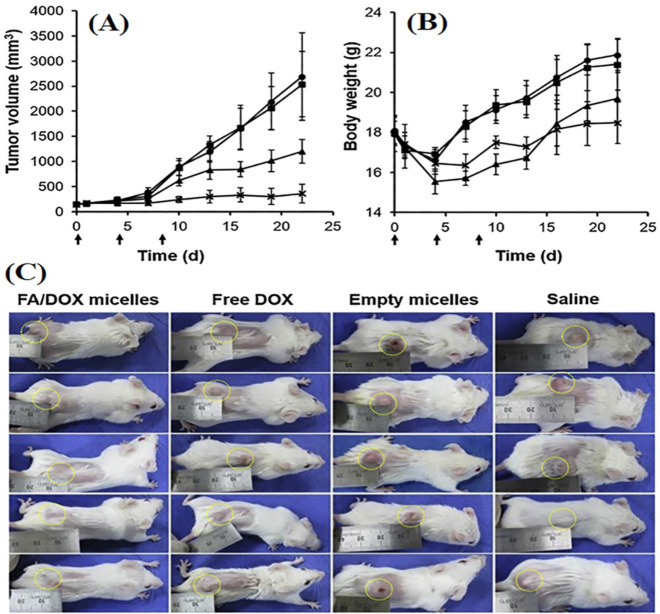
Image showing the tumor growth inhibition and impact on bodyweight of female Balb/c mice with MDR breast tumor (*n* = 5 ± SE) upon various treatment. Tumor-bearing mice were intravenously treated with normal saline (●), Pluronic (■), pure DOX (▲), and FA/DOX micelles (**_Χ_**) (dose of DOX = 4 mg/kg) at days 0, 4, and 8 (indicated by arrows). It indicated changes in (**A**) tumor volume; (**B**) body weight and (**C**) image showing MDR breast tumor-bearing Balb/c mice. The photographs of all the treated tumor-bearing mice were taken after 22 days of treatment. The tumors are indicated with yellow dotted circles. Reproduced with permission of Nguyen et al., Int. J. Pharm, published by Elsevier, 2015 [86].

Folate conjugated curcumin embedded nanostructured lipid carriers (FA-Cur-NLCs) showed significantly higher inhibition of tumor growth compared to curcumin NLCs and free curcumin solution in preclinical studies with MCF-7 tumor-bearing tumor xenografted Balb/c-nude mice model [85]. In another study, DOX-loaded FA-conjugated pluronic micelles significantly raised cellular uptake in MCF-7/MDR cells than untargeted micelles. It was also found that FA conjugated micelles showed ~3.3 and 8 folds higher reduction in tumor volume in 3 weeks in xenograft of MDR tumor-bearing Balb/c mice compared to untargeted micelles and free DOX solution, respectively (Figure 5) [86]. Gunduz et al. fabricated FA conjugated idarubicin-loaded magnetic NPs, which showed 90 folds higher uptake in MCF-7 cell lines compared to free idarubicin solution [87].

Biodegradable FA conjugated deoxycholic acid-O-carboxymethylated chitosan NPs developed by Wang and coworkers showed significantly higher cellular uptake than untargeted NPs in MCF-7 breast cancer cells. It was also found that FA decorated NPs showed a significantly higher pro-apoptotic effect in MCF-7 cells due to overexpression of FRs on the surface of MCF-7 cells, which considerably increased the uptake of NPs through FR-mediated endocytosis [88]. Vincristine sulfate-loaded PLGA–PEG NPs decorated with FA designed and characterized by Chen et al. exhibited significantly higher cellular uptake than NPs without the FA decoration. PLGA–PEG–FA NPs exhibited 1.52- and 3.91-folds higher cytotoxicity on MCF-7 cells than that of NPs without folate and free vincristine sulfate, respectively [89].

FA conjugated nanocarriers are one of the most explored nanomedicines, investigated for delivery of the therapeutic agent, imaging agent as well as theranostic agents for treatment and diagnosis of cancer. The above studies demonstrated that folate-targeted nanomedicine could provide a promising strategy for the effective and precise treatment of breast cancer.

### 4.3. Targeting of Estrogen Receptor

Estrogen receptors (ER) belong to the nuclear hormone receptor superfamily, which is a class of transcription factors regulated by small ligands. These receptors are differentially overexpressed to 60–80% in breast cancer cells [90]. Estrogens are internalized into the cell after binding to ER receptor, therefore estrogens can be explored as a ligand for targeting tumor cells overexpressing ER receptors [91,92].

**Figure 6:**
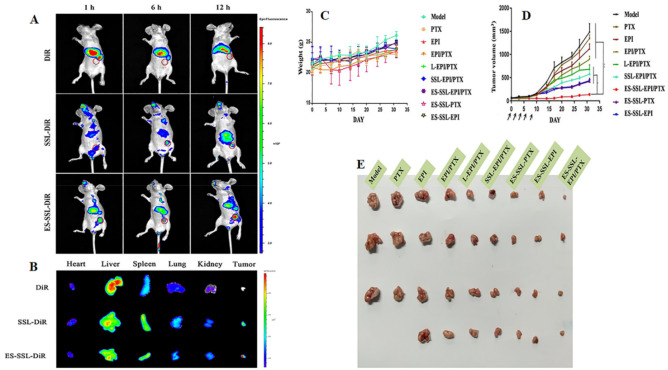
The photomicrograph showing the imaging of MCF-7 tumor-bearing Balb/c nude mice after intravenous administration of free DiR and DiR-labeled liposomes. (**A**) The in vivo imaging of Balb/c nude mice at predetermined time points; (**B**) The ex vivo imaging of different organs of Balb/c mice that were dissected just after 12 h of intravenous administration. The antitumor effect of ES-SSL-EPI/PTX in MCF-7 tumor-bearing Balb/c nude mice (*n* = 4). (**C**) Impact on body weights upon treatment; (**D**) Tumor volume after intravenous administration of free drugs and liposomal formulations; (**E**) The photomicrographs of MCF-7 tumors after intravenous administration of free drugs and liposomal formulations. ‘*’, ‘**’ represents the comparison with other treated groups, *p* < 0.05 and *p* < 0.01 respectively. Reproduced with permission of Tang et al., Int. J. Pharm, published by Elsevier, 2019 [93].

Estrone-targeted PEGylated paclitaxel and epirubicin co-loaded liposomal NPs showed a significantly higher cellular uptake, accumulation, and prolonged circulation time in both in vitro and in vivo investigations. Moreover, it was noticed that a significant suppression in the tumor growth was observed in animal model with no significant toxicity (Figure 6) [93].

In another research, tamoxifen, an ER antagonist, surface-grafted liposomes loaded with DOX showed enhanced cellular and nuclear uptake of DOX in comparison to DOX-loaded liposomes and free DOX solution. In vitro investigations demonstrated that tamoxifen and DOX co-loaded liposomes were more cytotoxic to ER overexpressing MCF-7 cells as compared to DOX liposomes, DOX solution, and tamoxifen-DOX solution. Tamoxifen-DOX liposomes exhibited remarkably increased inhibition of tumor growth in animal model compared to DOX solution and DOX liposomes [94].

Estrone decorated DOX-loaded stealth liposomes exhibited significant cytotoxicity, accumulation, and antitumor efficacy compared to untargeted formulations in ER-positive MCF-7 tumor-bearing female Balb/c-nude mice [95]. In a previous study, tamoxifen-loaded poly (ethylene glycol)-thiol gold nanoparticle exhibited significantly higher cellular uptake and cytotoxicity in ERα(+) human squamous oral cancer HSC-3 and MCF-7 breast cancer cells compared to untargeted NPs [96]. Similarly, estrone decorated liposomes showed higher accumulation in the ER expressed breast tissue compared with the plain drug and conventional liposomes [91].

These studies demonstrated that estrone-targeted nanomedicine may advance the treatment of various estrogen receptor overexpressing breast cancers by utilizing receptor-mediated targeting of anticancer agents.

### 4.4. Targeting of CD44 Receptor/Hyaluronan Receptor

The glycosaminoglycan hyaluronan is a naturally occurring component of the extracellular matrix. Hyaluronic acid (HA) plays a crucial role in cell proliferation, migration, and invasion, and as these processes are involved in inflammation and cancer progression, therefore HA is significant in cancer pathophysiology. The HA receptor CD44 is found sparsely on the surface of epithelial, hematopoietic, and neuronal cells, and is abundantly overexpressed in various cancer cells [97,98,99]. CD44 regulates lymphocyte adhesion to endothelial cells during lymphocyte migration [100], a process that is comparable to solid tumor metastasis [101]. It is also involved in the regulation of the proliferation of cancer cells [102]. Recently, the cancer stem cell theory has proposed that CD44 can be employed as a marker of breast cancer stem cells [103,104]. The relationship between tumor cells and HA receptors indicates that it may be possible to exploit HA for active targeting to tumor cells bearing this receptor [105,106].

A novel redox-responsive and HA-functionalized chitosan lipoic acid NPs (HA-CSLA-NPs) loaded with cytoplasmic 17α-methyltestosterone showed significantly higher cellular internalization through CD44 receptors with rapid drug release, and enhanced cytotoxicity against CD44 overexpressing BT-20 breast cancer cell line as opposed to CD44 negative MCF-7 cell line [107]. Previously, Cerqueira and coworkers developed HA-modified PLGA NPs, which showed significantly enhanced cellular uptake and cytotoxicity against MDA-MB-231 breast cancer cells compared to non-HA-modified NPs. [108]. Liu et al. designed HA-decorated NLCs for co-delivery of baicalein and DOX for breast cancer therapy. In vitro cytotoxicity assay against MCF-7/ADR cells exhibited 2.25 and 12-fold reduction of IC_50_ value with HA decorated NLCs in comparison to baicalein and DOX loaded NLCs, and baicalein and DOX solution, respectively. In vivo antitumor efficacy study using Kunming mice bearing MCF-7/ADR breast cancer cells xenograft showed higher inhibition in tumor growth with targeted NLCs as compared to untargeted baicalein and DOX loaded NLCs, baicalein NLCs, DOX NLCs, and baicalein and free DOX solution [109].

Zhong et al. developed HA L-lysine methyl ester-lipoic acid (HA Lys-LA) NPs. These HA Lys-LA NPs exhibited 20-folds higher tumor uptake of DOX compared to free DOX. DOX-loaded crosslinked HA-Lys-LA NPs possessed significantly higher targetability and excellent antitumor activity towards CD44 positive MCF-7/ADR cells. Moreover, the developed NPs exhibited significantly greater survival of mice throughout the experimental period of 44 days and showed lesser side effects compared to untargeted NPs and free drugs [110]. In another study, Zhao et al. developed HA matrix NPs with intrinsic-CD44-tropism loaded with rapamycin which showed 3.2-fold drug uptake in CD-44 positive MD-MB-468 breast cancer cells compared to the free rapamycin. The study outcomes in vivo pharmacokinetics showed that HA decorated NPs exhibited a 2.96-fold increase in area under the curve (AUC) than that of the free drug, and the concomitant total body clearance was 8.82-fold slower than free drug [111].

Docetaxel-loaded self-assembled NPs of PLGA/HA block copolymers exhibited significantly higher uptake, enhanced cytotoxicity in MDA-MB-231 cells by CD44-mediated endocytosis compared to untargeted NPs and free drug. In vivo studies with Balb/c nude mice showed that HA decorated NPs exhibited significantly improved cancer targeting and anticancer activity compared to untargeted NPs and free drug [112]. Yang et al. developed HA oligosaccharide lipid NPs loaded with paclitaxel, which exhibited significantly enhanced antitumor response and activity of chemotherapies both in vitro and in vivo compared to nontargeted NPs [113].

These studies suggest that HA could be employed as a promising targeting ligand to target various nanomaterials carrying the anticancer payload to the CD44 receptor for the treatment of breast cancer.

### 4.5. Targeting of LHRH Receptor

Luteinizing hormone-releasing hormone (LHRH) is a hormonal decapeptide produced by the hypothalamus. It is also known as Gonadotropin-releasing hormone (GnRH). It plays an important role in the regulation of the pituitary-gonadal axis and reproduction. LHRH receptors are overexpressed in 80% of endometrial and ovarian, 86% of prostate, 50% of breast, and 80% of renal cancers [114]. These LHRH receptors are not expressed significantly in normal organs, therefore LHRH can act as a targeting ligand to improve the cellular uptake of anticancer drugs to LHRH receptor-positive cancerous cells like breast cancer cells and reduce the peripheral side effects [115].

Recently scientists conjugated LHRH with anticancer drugs, prodigiosin or paclitaxel, separately and evaluated these LHRH drug conjugates for treatment and specific targeting in MDA-MB-231 TNBC cells and tumor-bearing female nude mice. The designed conjugates showed promising efficiency to target TNBC cells specifically with the ability to cause significant regression in tumor growth without any significant toxicity [116]. Previously, scientists investigated the cellular uptake efficiency of LHRH-conjugated PEG-coated magnetite NPs (LMNPs) and PEG-coated magnetite NPs (MNPs) in normal breast cells and TNBC cells, and normal breast cells. In the result of this study, scientists observed enhanced uptake for LMNPs into TNBC cells attributed to the presence of LHRH on the surface. The entry of nanoparticle into breast cancer cells are also explored using a combination of thermodynamics and kinetics models [117]. The concepts of thermodynamics and kinetics models explain the interaction between a ligand decorated nanoparticulate system and receptors expressed over the cellular membrane. During the receptor-mediated endocytosis of the nanoparticulate system, cellular receptors bind to the ligands present on the nanoparticulate system to decrease the free energy of the system. It is proved in the current investigation that receptor-mediated endocytosis involves an interplay between thermodynamics and kinetics.

Varshosaz et al. developed magnetic LHRH chitosan bioconjugated NPs which demonstrated significantly enhanced cellular uptake and a 2-fold reduction in IC_50_ value against LHRH overexpressing MCF-7 cells compared to nontargeted NPs [118]. In another study, Li et al. developed cisplatin-loaded LHRH-modified dextran NPs which exhibited significantly higher uptake of cisplatin and cytotoxicity with a significant reduction in nephrotoxicity of cisplatin compared to nontargeted NPs. LHRH modified NPs exhibited a higher antitumor effect with low systemic toxicity compared to untargeted NPs in antitumor efficacy study in Balb/c mice bearing 4T1 tumors [119]. Furthermore, Taheri et al. developed LHRH conjugated methotrexate-human serum albumin NPs which exhibited about 7-fold higher antitumor efficacy compared to untargeted NPs. Additionally, LHRH NPs exhibited 216.66% improvement in the life span of mice [120].

The studies discussed above supports further exploration of LHRH conjugated nanomedicine for treatment and diagnosis of breast cancer including its most complicated version, TNBC. Thus, LHRH can be used as a potential targeting ligand for the targeted treatment of LHRH overexpressed breast cancers.

### 4.6. Targeting of Transferrin Receptor

Transferrin (Tf) is a serum nonheme iron-binding blood plasma glycoprotein responsible for the transport of ferric ions (Fe^3+^) through TfRs on the plasma membrane. The delivery of Fe^3+^ is mediated via clathrin-mediated endocytosis and the TfRs recycle back to the cell surface. TfRs are overexpressed in cancer cells owing to their higher necessity of iron and there exists a correlation between the number of TfRs expressed and the proliferative ability of the tumors [121,122]. The TfR expression on tumor cells is about 10-fold greater than on normal cells. Tf specifically binds to TfR and represents an appropriate ligand for accomplishing site-specific targeting. Tf has a high specificity of endocytic uptake by TfR. This receptor can be targeted in two ways: i) for the delivery of anticancer therapeutics into breast cancer cells or ii) to block the natural function of the receptor leading to cancer cell death. Tf-decorated carrier systems have been widely used for the intracellular targeting of tumor cells including breast cancer cells via receptor-mediated endocytosis [123]. The overexpression of TfR on breast cancer cells, their ability of cellular internalization, as well as iron need for proliferating cells make TfR a promising target for targeted therapy in breast cancer.

Venkatesan et al. developed DOX loaded redox responsive Tf-capped mesoporous silica nanoparticles with PEG surface modification (DOX-MSN-Tf@PEG) to increase internalization in MCF-7 breast cancer cells via Tf/TfR mediated endocytosis. In vivo studies showed that there was a significant inhibition in tumor growth and reduced side effects through cell apoptosis as determined by TUNEL assay [124]. Cui et al. developed Tf decorated DOX and curcumin co-loaded pH-sensitive self-assembled NPs (Tf-PEG-Curcumin/DOX NPs), which demonstrated a significant increase in cellular uptake and cytotoxicity in TfR positive MCF-7 breast cancer cells. In vivo antitumor efficacy study in MCF-7 breast tumor-bearing female Balb/c mice, Tf conjugated NPs showed efficient and strong antitumor efficacy as compared to that of nontargeted NPs [125]. Tf functionalized nutlin-3a encapsulated PLGA NPs surface-functionalized with Tf ligand exhibited 22- and 3-folds higher uptake than native nutlin-3a and nonconjugated NPs, respectively, in MCF-7 breast cancer cells. The conjugated system exhibited increased activation of the p53 pathway in comparison to native therapeutics at the molecular level and observed increased cell cycle arrest and apoptosis [126].

Tf has also been conjugated with lipid-coated PLGA NPs carrying the aromatase inhibitor, 7α-(4′-amino) phenylthio-1,4-androstadiene-3,17-dione (7α-APTADD) which exhibited significantly higher drug uptake in SK-BR-3 breast cancer cells compared to untargeted NPs. Moreover, Tf-decorated NPs exhibited a 2.8-fold reduction in IC_50_ value compared to untargeted NPs [127]. In another study, Mulik et al. developed Tf-decorated curcumin encapsulated solid lipid nanoparticles (SLNs), which showed improved photostability, apoptosis, and anticancer activity against MCF-7 cells. The improvement in cell death with Tf-SLNs was about 3 and 1.5-fold greater in comparison to curcumin solution and untargeted SLNs, respectively [128].

Recently, Li et al. developed Tf-decorated piperine-loaded polymeric nanoparticles for targeted treatment of breast tumors [129]. In vitro cell culture studies revealed that the developed nanoparticles exhibited significantly enhanced cellular uptake and cytotoxicity against different breast cancer cells. Targeted nanoparticles exhibited 2.35- and 1.2-times higher cytotoxicity in MDA-MB-231 and 4T1 breast cancer cells respectively compared to the nontargeted nanoparticles. Furthermore, in vivo efficacy study in Female BALB/c mice bearing 4T1 breast tumor revealed much better antibreast cancer efficacy of TfRs targeted nanoparticles compared to the nontargeted nanoparticles.

Thus, a nanoparticulate system can be successfully employed for TfR targeted treatment of breast cancers using Tf as a targeting ligand.

### 4.7. Targeting of Integrin Receptor

Integrins represent the family of heterodimeric integral membrane proteins encompassing not less than 24 combinations of 18α and 8β subunits that mediate cell adhesion. Integrin receptors are intricately involved in cell–cell and cell–extracellular matrix interactions, cell adhesion, migration, survival, growth, cytoskeleton organization, and cell signaling. In cancer, integrins stimulate the proliferation of tumor cells and tumor vascular endothelial cells [130]. Integrins contribute significantly to distinct phases of metastasis, including tumor cell binding, invasion, growth, and angiogenesis [131]. These receptors are overexpressed in breast cancer cells. Therefore, they can be considered as potential targets for receptor-mediated delivery of therapeutics in breast cancer [132].

Researchers have designed leukocyte-mimicking nanovesicles, i.e., leukosomes, for effective delivery of DOX for the treatment of breast cancer and melanoma. They observed significantly higher tumor accumulation of leukosomes with reduced tumor volume and prolonged survival span and thus, more potent anticancer activity in comparison to free DOX solution. There was a significant inhibition in tumor growth with DOX-loaded leukosomes as compared with free DOX in both breast and melanoma tumors [133]. Shroff and Kokkoli, developed fibronectin-mimetic peptide grafted DOX-loaded PEGylated NPs targeted to α5β1-expressing MDA-MB-231 breast cancer cells. Their results showed that the targeted NPs specifically binds to integrin α_5_β_1_, thereby providing a tool to target α_5_β_1_-expressing cancer cells in vitro as well as in vivo. In vitro binding efficacy study showed that functionalized PEGylated liposomes exhibited enhanced binding as compared to the untargeted PEGylated liposomes, and their binding efficiency increased with increasing fibronectin-mimetic peptide concentration. Furthermore, functionalized PEGylated liposomes exhibited higher cytotoxicity to MDA-MB-231 cells compared to untargeted PEGylated liposomes [134]. Graf et al. fabricated cyclic pentapeptide c(RGDfK)-conjugated cisplatin prodrug-encapsulated PLGA-PEG NPs to target α_v_β_3_ integrins on cancer cells. The conjugated NPs exhibited significantly higher uptake in comparison to untargeted NPs in MCF-7, MCF7-MFP1, DU145, DU145LN2, PC3, PC3MLN4 cell lines. In vivo cytotoxicity study in female nude mice xenograft bearing MCF-7-MFP1 breast cancer cells showed that RGD-targeted cisplatin encapsulated NPs exhibited significantly higher cytotoxicity compared to nontargeted NPs [135].

From the studies discussed here, we could conclude that integrin receptor-targeted nanomedicine could be explored as a promising therapeutic intervention for breast cancer treatment.

### 4.8. Targeting of Vasoactive Intestinal Peptide (VIP) Receptor

Vasoactive intestinal peptide (VIP) is a 28-amino acid neuropeptide of the glucagon-secretin class which is pervasive in both the central and peripheral nervous systems. VIP receptors are five times more profuse in breast cancer cells than in normal breast cells [136]. The VIP receptor offer to be a crucial molecular target for breast cancer owing to its prodigious abundance. The overexpression of VIP receptors is largely exploited to develop novel paradigms of breast cancer therapeutic intervention [137].

Pirarubicin, an anticancer drug, which is an analog of the anthracycline antineoplastic antibiotic DOX and causes serious side effects, was loaded into sterically stabilized micelles of 1,2-Distearoyl-sn-glycero-3-phosphoethanolamine-N- methoxy-- polyethylene glycol 2000 (DSPE-PEG2000). Furthermore, these micelles were surface-functionalized with VIP to target breast cancer cells. In the in vitro studies with MCF-7 breast cancer cells as well as in nude mice xenograft model of 4T1 breast cancer, these VIP functionalized pirarubicin-loaded DSPE-PEG2000 micelles showed higher anticancer activity without significant toxicity [138]. Cancer stem cells (CSCs) are considered to play important role in tumor progression and regeneration and considering the association of VIP overexpression with CSCs, Gulçur et al. designed a curcumin-loaded nanomedicine surface-functionalized with VIP to treat breast cancer. In the result of this study, the scientist observed that VIP conjugated curcumin nanomedicine showed a significant reduction in IC_50_ up to 14.2 ± 1.2 μM in comparison that forms free curcumin which was 26.1 ± 3.0 μM. Furthermore, this nanomedicine also showed inhibition in the formation of tumorsphere up to 20% at a dose of 5 μM [139].

Paclitaxel-loaded VIP surface grafted sterically stabilized phospholipid nanomicelles were designed by Dagar and coworkers to target VIP receptors. This VIP receptor-targeted nanomedicine exhibited a 2-fold improvement in ED_50_ value compared to free paclitaxel. Furthermore, the in vivo studies in female Sprague Dawley rats demonstrated that there was a significantly higher accumulation of paclitaxel delivered in the targeted carrier in the breast tumors due to its size, long circulation, and interaction with target receptors. A minimal accumulation of paclitaxel in healthy tissues particularly those associated with systemic toxicities (such as bone marrow) owing to target-specific delivery was observed [140]. Onyuksel et al. developed VIP-grafted paclitaxel-loaded sterically stabilized mixed phospholipid nanomicelles, which significantly inhibited MCF-7 cancer cell growth in a dose-dependent manner. Both targeted and untargeted formulations were ~7-fold more potent than solvent dissolved neat paclitaxel. VIP grafted nanomicelles were significantly more effective in drug-resistant BC19/3 breast cancer cells, compared to paclitaxel nanomicelles without VIP and paclitaxel dissolved by approximately 2- and 5-fold, respectively [141]. In another study, Onyuksel et al. developed 17-allylamino-17-demethoxy geldanamycin (17-AAG) loaded human VIP surface-grafted sterically stabilized phospholipid nanomicelles, which showed significantly greater cytotoxic to MCF-7 cells than that of untargeted nanomicelles [142].

From the above studies, it could be concluded that VIP can be utilized promisingly for receptor-specific targeted treatment of breast cancers including CSCs enriched breast cancers.

### 4.9. Other Receptors Targeted for Breast Cancer Therapy

Scientists have also explored some other receptors targeted nanomedicine for improving the treatment protocol of the world’s most reported cancer, breast cancer, in women. Tian et al. developed N-acetyl-D-glucosamine (NAG) decorated DOX loaded poly (styrene-alt-maleic anhydride)_58_-b-polystyrene_130_(P(St-alt-MA)_58_-b-PSt_130_) NPs, which showed that NAG conjugation enhanced the internalization and targeting ability of NAG-P(St-alt-MA)_58_-b-PSt_130_ NPs. In vitro anticancer activity in MCF-7 cells showed that DOX loaded NAG-P(St-alt-MA)_58_-b-PSt_130_ NPs exhibited an enhanced anticancer activity compared to NPs without NAG, which could be attributed to NAG receptor-mediated endocytosis [143]. Guo et al. modified the surface of DOX-loaded liposomes with a homing peptide with a sequence of YSAYPDSVPMMSK (YSA) having a high and specific binding affinity towards EphA2, which is a transmembrane receptor tyrosine kinase and is overexpressed on tumor cells as well as on tumor vasculature. The YSA decorated DOX-loaded liposomes exhibited significantly enhanced uptake and cytotoxicity in MDA-MB-231 TNBC cell lines and human umbilical vein endothelial cells (HUVEC). Therapeutic efficacy studies of YSA decorated DOX liposomes in Balb/c nude mice bearing MDA-MB-231 breast tumor xenografts showed significantly higher efficacy with low systemic and cardiac toxicity for EphA2-overexpressing metastatic breast cancer compared to nontargeted liposomes [144].

A ligand-gated ion channel, Alpha7 nicotinic acetylcholine receptor (α7 nAChR), is being investigated by scientists for targeted delivery of anticancer drugs due to its specific expression in cancer. In an attempt to target α7 nAChR, Mei et al. developed paclitaxel loaded and α-conotoxin ImI (it is a disulfide-rich toxin which has shown a strong affinity for α7 nAChR) conjugated poly (ethylene glycol) -(distearoyl-sn-glycero-3-phosphoethanolamine) (PEG_2000_-DSPE_800_) micelles which showed much higher cellular uptake, cytotoxicity, and apoptosis than that of unmodified micelles for either A549 or MCF-7 cells. In vivo antitumor efficacy study in MCF-7 tumor-bearing female nude mice demonstrated that targeted micelles showed significantly enhanced suppression of tumor growth during the experiment up to 18 days compared to untargeted micelles and Taxol [145].

Nishikawa et al. developed an antihuman heparin-binding EGFR monoclonal antibody targeted DOX encapsulated immunoliposomes which exhibited significantly higher uptake in Vero-H cells and MDA-MB-231 human breast cancer cells over untargeted immunoliposomes. Furthermore, DOX encapsulated and antiheparin-binding EGFR antibody-decorated liposomes caused strong suppression and regression of MDA-MB-231 tumors in Balb/c nude female mice [146].

Similarly, Taheri et al. developed biotin-conjugated methotrexate loaded human serum albumin NPs to target biotin receptors for the treatment of breast cancer. The in vivo anticancer activity in female Balb/c mice bearing 4T1 breast carcinoma demonstrated that biotin-targeted methotrexate NPs had significantly higher antitumor activity and reduced toxic effects than untargeted NPs and free drug. Furthermore, biotin-targeted methotrexate NPs increased the survival of tumor-bearing mice to 47.5 ± 0.71 days and increased their life span by 216.7%. In vivo studies in mice treated with biotin-targeted NPs showed weight loss of 8% in the body 21 days after treatment, whereas untargeted methotrexate NPs treatment at the same dose caused a weight loss of 27.05 ± 3.1% [147].

Yu et al. developed K237-(HTMYYHHYQHHL) peptide conjugated paclitaxel loaded aldehyde PEG–PLA NPs exhibited significantly higher uptake compared to untargeted NPs and free Taxol. The pharmacokinetic study showed that K237 conjugated paclitaxel-loaded NPs and unconjugated paclitaxel NPs exhibited an extended half-life of paclitaxel from 0.9 h to 8.77 h and 8.94 h, respectively compared to Taxol. Meanwhile, the AUC improved about 30-fold for K237 conjugated paclitaxel-loaded NPs and 27-fold for unconjugated NPs compared to Taxol. K237 conjugated paclitaxel-loaded NPs also exhibited significantly stronger antitumor efficacy compared to unconjugated NPs in female Balb/c nude mice bearing MDA-MB-231 breast tumors [148].

The prognosis of receptor-mediated targeted therapy in breast cancer is mainly dependent upon the extent of overexpression of the breast cancer-specific cellular receptors particularly human epidermal growth factor receptor-2 (HER2/Neu/ErbB2), estrogen and progesterone receptors. Triple-negative breast cancers (TNBC), which do not express any of these targets, i.e., estrogen receptor, progesterone receptor, and HER2 receptor, are the most challenging to treat as these show a poor response to both HER2 targeted and hormonal receptor (estrogen, and progesterone receptor) targeted therapies. Theranostic nanomedicines are emerging as a novel paradigm with the ability for concurrent delivery of imaging (with contrasting agents), targeting (with biomarkers), and anticancer therapeutics through the single delivery vehicle as cancer theranostics. It can transpire as a promising strategy to overcome various limitations for effective management of breast cancer including its most aggressive form, TNBC [149]. Recently, Mu Q et al. developed a theranostic nanomedicine system for targeted chemo-immunotherapy in TNBC. The developed formulation system remarkably inhibits tumor growth and metastasis and substantively prolongs mean survival in a mouse model of TNBC [150].

The potential of different receptor-targeted nanomedicines explored for the treatments of breast cancer is concisely summarized in Table 1. We could conclude that nanomedicine may improve the therapeutic outcome in the case of breast cancer treatment which could further be precise with the aid of receptors.

## 5. Clinical Translation and Future Challenges

Owing to the significant advances in the field of nanobiotechnology, multiple nanomedicines-based anticancer therapeutic agents are now available in the market and accessible for clinical use, and some of the products are in different stages of clinical trials. Most of the anticancer nanomedicine available in the market or under clinical trials utilized previously approved active therapeutic moieties encapsulated into different nanocarriers including liposome, polymeric micelles, dendrimers, nanoemulsion, and inorganic nanoparticles [197]. Despite the growing arsenal of nanomedicine-based drug delivery systems currently liposomal formulation is dominant in the nanomedicine market [197,198]. Likewise, various complex nanoproducts have been approved and commercialized for the treatment of various forms of cancer and numerous nanomedicines are progressing to clinical trials every year. Liposomal formulations account for the majority of modified nanomedicine under clinical investigation. So far, the most frequently observed therapeutic benefit of marketed nanomedicine has been reduced toxicity with enhanced efficacy. Recently approved VYXEOS^™^, a liposomal formulation of daunorubicin and cytarabine in several countries, including the USA and EU against acute myeloid leukemia (t-AML) or AML with myelodysplasia-related changes demonstrated remarkably improved survival and response rates, with tolerable toxicity in older patients [197,199].

Even after obtaining promising results in preclinical investigations, there are still numerous obstacles in development of targeted cancer nanomedicines that restrict its success in clinical settings. The various biological and translational barriers are being identified. These barriers need to be overcome in order to improve the clinical outcomes of targeted therapies. Among various challenges, the greatest is the production of targeted nanoparticles at a large scale since it faces continuous issues such as multistep synthetic procedure, higher manufacturing cost, and reproducibility of biopharmaceutical attributes [200,201,202]. Even though nanoparticle-drug conjugates turned out to be safe in preclinical studies, the possibility of toxicity at a particular dose of a drug, dosage form, and administration route should be assessed rigorously before proceeding to investigation in humans. Furthermore, the assortment of model receptors and apt ligands for the coherent fabrication of receptor-targeted nanomedicines poses another challenge in its clinical translation [203,204,205]. Cancer development might also be triggered in the case of targeted therapies. Targeted delivery of vitamin B12 significantly reduces the risk of cancer progression via enhanced methylation. Cancer cells are not the only cells where folic acid is overexpressed; it is also highly expressed in the normal cells of the placenta, lungs, choroid plexus, and lungs, which might cause irregular drug distribution that can result in systemic toxicity. To resolve the aforementioned limitations, profound knowledge of cancer heterogeneity, the molecular intervention of tumor progression, and insights into tumor metastasis, which may be vital in selecting the proper therapy for the right patients, are required. The stability of nanoparticles, scalability, cost-effectiveness, batch-to-batch reproducibility, and precision in the fabrication of nanoparticles should be considered to enhance clinical performances. For selecting ideal target receptors, their expression and kinetics patterns in patients, the safety of ligands, and exhaustive screening of receptor types must be closely monitored to augment the clinical efficacy of receptor-targeted nanomedicines in breast cancer alleviation.

## 6. Conclusions

In a nutshell, receptor-targeted modified nanomedicines foster a great prospect of optimal therapeutic and drug delivery approaches. Modified nanomedicine possesses the remarkable potential to address the shortcomings of the conventional chemotherapy approach. Strenuous research on tumor milieu, pathophysiology, and progression stemmed exploration of novel propitious targets for breast cancer alleviation. In the interim, substantial improvement in the fabrication of various nanoparticulate systems and insights of their physicochemical and pathophysiological obstacles made them a promising targeting system cognizant for fixing the clinical effect of nanomedicine for breast cancer therapeutic intervention. Various advancements in nanomedicines such as stimuli-responsive nanoparticulate systems were developed until now to deliver anticancer agents for treating breast cancer. Several ligands were utilized for specific targeting of receptors that are overexpressed in breast cancer cells. The findings of the preclinical investigation established improved cellular internalization, mitigated toxicity issues, and multidrug resistance in breast cancer cells through a targeted nanoparticle–drug conjugation/encapsulation approach. Such encouraging outcomes propel the clinical translation of receptor-targeted surface-modified nanomedicines. Nonetheless, still, many more arduous investigations are highly needed for selecting the ideal target receptor and for exploring the ligand–receptor interaction.

## Figures and Tables

**Table 1:** Receptor-targeted surface-modified nanomedicine for the treatment of breast cancer.

Receptor Targeted	Ligand	Type of Nanomedicine	Drug Loaded	Breast Cancer Model	Outcome	Ref.
HER2	Herceptin and PEG	Carbon Based NPs (CNP)	DOX	SK-BR-3 and MDA-MB-231 cells	High cellular uptake with low toxicity Significant inhibition of antitumor activity both in vitro and in vivo	[151]
	Anti-HER2 monoclonal antibody	Core-shell chitosan NPs	DOX	MCF-7 cells	In vitro cytotoxicity studies showed the lowest IC_50_, Increased therapeutic efficacy of DOX	[152]
	Trastuzumab	Gold NPs	-	HER2 overexpressing human ovarian SKOV-3 cell line	In vitro biological studies indicated higher affinity and cytotoxicity towards the SKOV-3 cells.	[153]
	Trastuzumab	Polymeric NPs	Emtansine (DM1)	MDA-MB-453 xenograft bearing mice	Inhibition of tumor growth by 88%, Fewer toxic effects in vivo.	[154]
	Herceptin	Polyethyleneimine (PEI)/PLA NPs	DOX	Xenograft nude mice bearing SK-B In vivo R-3 cancer cells Female Balb/c mice	-Superior cytotoxic effect in the cancer cells, Cellular internalization via receptor-mediated endocytosis, and effective targeting ability of the tagged moiety. -Reduced side effects of DOX in the xenograft tumor model.	[155]
	Trastuzumab	β-1,3-glucan (Glu) succinate NPs	DOX	In vitro/MCF-7 and HER2 positive murine (4T1) breast cancer cell line In vivo/female Balb/c mice bearing 4T1 breast tumor	Significantly higher cellular uptake Significantly improved in vitro and in vivo cytotoxicity	[156]
	Herceptin	PLA NPs	TPGS	SK-BR-3 cells	-Targeted NPs exhibited significantly higher cellular uptake compared to nontargeted NPs, -Targeted NPs exhibited a 9.6-fold lower IC_50_ value compared to nontargeted NPs	[69]
	Herceptin	Micelles	Docetaxel, polo-like kinase 1 siRNA	NIH3T3, MCF-7, and SK-BR-3 breast cancer cells	Significantly enhanced internalization into the cytoplasm of the SK-BR-3 cells, excellent reduction in IC_50_ value	[157]
	Herceptin	poly (ε-caprolactone), PLA, PLGA, polyester urethanes (PURs), and N-Boc-serinol NPs	Paclitaxel	In vitro/HER2 than MCF-7 breast cancer cell lines	1.2- and 1.3-fold higher cellular uptake compared to nontargeted NPs Significantly higher cytotoxic effect compared to non-HER coated NPs	[158]
	Trastuzumab	PEG-PLGA NPs	Docetaxel	In vitro/SK-BR-3 and BT-474 cell lines	Significantly higher cellular uptake Improved in vitro cytotoxicity	[159]
	HER2 antibody	Glycerol monooleate coated magnetic NPs (GMO-MNPs)	paclitaxel and/or rapamycin	In vitro/human breast carcinoma MCF-7 cell line	Significantly enhanced cellular uptake Targeted paclitaxel-loaded GMO-MNPs exhibited ~24 folds lower IC_50_ value than native paclitaxel and ~3 folds lower IC_50_ value than nontargeted paclitaxel loaded GMO-MNPs. In the case of rapamycin drug, the targeted GMO-MNPs exhibited ~71 folds lower IC_50_ value than native drug and ~10 folds lower IC_50_ value than nontargeted GMO-MNPs. In the case of combined drug formulation, the targeted NPs exhibited ~55 folds lower IC_50_ value than native drugs and ~7 folds lower IC_50_ value than unconjugated NPs	[72]
	Fab’ fragments of a humanized anti-HER2 monoclonal antibody	PLGA NPs	PE38KDEL	In vitro/D2F2/E2 and SK-BR-3 cells In vivo/Balb/c mice bearing D2F2/E2 breast tumors	Significantly enhanced in vitro and in vivo cytotoxicity Reduced toxicity and immunogenicity	[160]
	Fab’ fragments of a humanized anti-HER2 monoclonal antibody	PLGA NPs	PE38KDEL	In vitro/BT-474, MDA-MB-231 and MCF-7 breast cancer cell lines In vivo/Balb/c nude mice bearing BT-474 human breast cancer cells	Exhibited notably enhanced cytotoxicity against all three cell lines compared to NPs without conjugated with antibody Improved in vivo antitumor activity compared to immunotoxin PE-HER Reduced systemic cytotoxicity	[161]
	Trastuzumab	PLGA/montmorillonite NPs	paclitaxel	In vitro/SK-BR-3 cells	Significantly higher cellular uptake 12.74 times higher cytotoxicity than that of the bare NPs and 13.11 times higher than Taxols	[74]
EGFR	Anti-EGFR Protein	Immunonanoparticles (INPs)	Paclitaxel	MDA-MB-468 TNBC cell line. Athymic mice model	INPs showed significantly enhanced cytotoxicity, i.e., remarkable reduction of cell viability NPs penetrated into the cell membrane and showed significantly enhanced cellular uptake. Significant reduction in the expression of EGFR protein	[162]
	CL4 aptamer	PLGA-b-PEG NPs	Cisplatin	MDA-MB-231 and MDA-MB-231 EGFR-KO cells; Female mice bearing MDA-MB-231 xenografts	High and fast cellular uptake in EGFR-positive TNBC cells as well as high cytotoxicity, i.e., decreased cancer cell viability Significantly high tumor targeting efficiency and therapeutic efficacy without any signs of systemic toxicity	[163]
	Anti-SPARC antibody	Human serum albumin NPs	Lapatinib	In vitro/4T1 cells (murine TNBC cells)	Targeted NPs (i.v.) effectively enhanced the accumulation of lapatinib in tumor tissue at 2.38 and 16.6- times the level of LS (i.v.) and Tykerb (p.o.), respectively	[164]
	Cetuximab	TPGS Micelles	Docetaxel	In vitro/MDA-MB-468 and MDA-MB 231 cancer cell line	Significantly higher cellular uptake 205.6- and 223.8-folds higher efficiency than Taxotere (free drug solution) for the MDA-MB-468 and MDA-MB-231 cell lines respectively	[70]
	NH2- CMYIEALDKYAC-COOH peptide	Peptide nanoconjugate	DOX	In vitro/SW480, SGC-7901, B16 and MCF-7 cancer cell lines In vivo/C57BL/6 mice bearing murine melanoma B16 tumor	Significantly higher cellular uptake Exhibited higher in vitro cytotoxicity in EGFR-overexpressed cancer cells Targeted NPs exhibited a significantly higher reduction in tumor volume compared to nontargeted NPs.	[165]
	EGFR-antibody	PLGA NPs	Rapamycin	In vitro/MCF-7 cell line	13-fold higher cellular uptake compared to nontargeted NPs Significantly higher antiproliferative activity than unconjugated rapamycin loaded NPs and native rapamycin	[73]
Folate	FA	SLNs	Letrozole	MCF-7 cancer cells	Significantly enhanced cytotoxicity Induction of caspase-3 dependent apoptosis	[166]
	FA	pH-responsive poly(β-thiopropionate) NPs with a magnetic core	DOX	MCF-7, BT549, and MD-MBA-231 cells. MCF-7 cells injected Female Balb/c nude mice	-FA-DOX@IONPs showed the strongest cytotoxicity against breast cancer cells Suppression in in vivo tumor growth in mice -No signs of toxicity in healthy organs	[167]
	FA	SPIONs	DOX	MCF-7 cell line Xenograft MCF-7 breast tumor of nude mice	-Internalization by receptor-mediated endocytosis -Inhibition in tumor growth -No significant toxicity of DOX@ FA-SPIONs on mice organs	[168]
	Folate	MSNs	DOX and Bcl-2 siRNA	MDA-MB-231 breast cancer cell line	significantly enhanced intracellular uptake Induction of remarkable cell apoptosis	[169]
	FA	PEG-poly (propylene succinate) NPs	Ixabepilone	HeLa Kyoto (HeLa K) and MCF-7 cells	Enhanced cellular uptake by receptor-mediated endocytosis	[170]
	Folate	Nanostructured lipid carriers	Curcumin	MCF-7 human breast cancer cells Balb/c nude mice	-3.52 and 10.41-fold reductions in IC_50_ values compared to nontargeted NLC and curcumin solutions, respectively, in vitro -Significantly higher tumor growth inhibition in vivo.	[85]
	Folate	Lipid-polymer hybrid NPs	Paclitaxel	EMT6 breast tumor cell line/Balb/c female mice	-Significantly higher cellular uptake, significantly improved antitumor efficacy in vitro and in vivo	[171]
	FA	Nanomicelle plexes of hydrophilic cationic star-block terpolymer	DOX and Bcl-2 siRNA	In vitro/MCF-7 breast cancer cell line	-Significantly higher cellular uptake Significantly higher in vitro cytotoxicity compared to nontargeted NPs	[172]
	FA	Polymeric micelles	DOX	MCF-7/MDR cells/Balb/c mice	Significantly higher cellular uptake, ~3.3 and 8-fold higher tumor volume reduction in 3 weeks in vivo compared to nontargeted micelles and free DOX solution.	[86]
	FA	Magnetic NPs	Idarubicin	MCF-7 cell line	2-fold higher tumor inhibition in vitro.	[87]
	FA	PLGA-PEG NPs	17-AAG	In vitro/MCF-7 human breast cancer cells	Much higher intracellular uptake 2-fold higher cytotoxicity compared to nontargeted NPs	[173]
	FA	PLGA-PEG NPs	Vincristine	In vitro/MCF-7 human breast cancer cells	Significantly higher cellular uptake Targeted NPs exhibited 1.52 and 3.91-folds higher cytotoxicity on MCF-7 cells than that of nontargeted NPs and free vincristine sulfate, respectively	[89]
Estrogen	Estrone	Liposomal NPs	Epirubicin and paclitaxel	MCF-7 cell line	Significantly improved accumulation in tumor cells, Increased systemic circulation time and biodistribution in main organs, Suppression in tumor growth without inducing toxicity.	[93]
	Raloxifene	Chitosan NPs	DOX	MCF-7 cell line	Significantly higher cytotoxicity Enhanced antitumor efficacy	[174]
	Estrone	Chitosan NPs	DOX	MCF-7 cell line Tumor-bearing rat model	Higher potency of developed NPs Significantly improved efficacy least toxic against blood cells and cardiac tissues hence reduction of cardiotoxicity of DOX	[175]
	Estrone	Liposomes	Mitoxantrone	HL-60 cells.	Specific cellular uptake via the ligand–receptor-mediated pathway Significant reduction in the growth of HL-60 cells	[176]
	Estrone	Gelatin NPs	Noscapine	ER-positive MCF-7 and ER-negative MDA-MB-231 breast cancer cell lines	Cell uptake study displayed higher accumulation of Nos-ES-GN in MCF-7 cells than that of MDA-MB-231 cells, Internalization by receptor-mediated endocytosis	[177]
	Tamoxifen	Liposomes	DOX	In vitro/MCF-7 breast cancer cell line In vivo/female Balb/c nude mice bearing MCF-7 breast tumor	-Significantly higher cellular and nuclear uptake -Significantly higher in vitro and in vivo cytotoxicity compared to nontargeted liposomes and free drug solution	[94]
	Estrone	Liposomes	DOX	MCF-7 breast cancer cell line, MDA-MB-231 cells/female Balb/c nude mice	13-fold higher half-life (t_1/2_) compared to free drug solution, 24.27 and 6.04-fold higher cellular uptake compared to free drug solution and nontargeted liposomes, respectively Significantly higher antitumor efficacy in vivo	[95]
	Tamoxifen (ER antagonist)	Gold NPs (AuNPs)	Tamoxifen	In vitro/MCF-7 breast cancer cell line	Significantly higher cellular uptake 2.7-folds improvement in drug potency than nontargeted NPs	[96]
CD44	HA	Redox-responsive HA–chitosan–lipoic acid NPs	17α-methyl testosterone	MCF-7 and BT-20 cell lines	Improved cellular internalization, Significantly enhanced cytotoxicity and apoptosis	[107]
	HA	HA NPs	Docetaxel	4T1-Luc breast cancer cells Subcutaneous 4T1-Luc tumor-bearing mice	Selective cellular uptake and remarkable cytotoxicity Enhanced growth and metastasis inhibition of 4T1-Luc breast tumors. Better antitumor, antimigration, and anti-invasion activity	[178]
	HA	Single-walled carbon nanotubes	DOX	MDA-MB-231 (human breast cancer) cell line	-Improved cellular uptake -Inhibition of migration of MDA-MB-231 cells -Inhibition of the growth of cancer cell spheroids	[179]
	HA	Polyethyleneimine (PEI)-PLGA NPs	TRAIL plasmid and Gambogic acid	MCF-7 and MDA-MB-231 breast cancer cell lines, Mouse mammary breast tumor 4T1 cell line Tumor-bearing nude Balb/c mice	-Selective uptake of the drugs in TNBC cells, -Apoptosis of TNBC cells both in vitro and in vivo -Significant inhibition in the growth of tumors	[180]
	HA	SLNs	Ibuprofen and Paclitaxel	CD44 negative BT-474 cell line and CD44 positive MDA-MB-231 breast cancer cell line	-Improvement in cellular uptake and induction of apoptosis. -Significantly higher inhibition of the growth of the MDA-MB-231 cells.	[181]
	HA	Nanostructured lipid carriers	Baicalein and DOX	MCF-7/ADR breast cancer cells/Kunming mice	-2.25 and 12-fold reduction of IC_50_ value compared to nontargeted NLCs and mixture of drug solution in vitro, significantly enhanced in vivo antitumor efficacy	[109]
	HA	NPs based on HA- L-lysine methyl ester- lipoic acid) conjugates	DOX	In vitro/MCF-7/ADR cancer cell line In vivo/MCF-7/ADR tumor-bearing nude mice	-20-folds higher cellular uptake compared to free drug -Excellent targetability and superior antitumor activity in vitro -Significantly greater survival of mice and low side effects in vivo	[110]
	CD44 monoclonal antibody	MSNs	DOX	In vitro/MCF-7/ADR1 cancer cell line In vivo/female nude mice bearing the resistant MCF-7/MDR1 tumors	-Significantly higher cellular uptake -Significantly higher tumor growth inhibition and induced apoptosis	[182]
	HA	Micelles	Paclitaxel and AURKA specific siRNA (si-AURKA)	MDA-MB-231 breast cancer cell line, Balb/c nude mice	Significantly enhanced cellular uptake and synergistic cytotoxic effect of drug and siRNA in vitro and in vivo	[183]
	HA	Chitosan NPs	DOX and miR-34a	In vitro/MDA-MB-231 cancer cells In vivo/female athymic nude Balb/c mice bearing MDA-MB-231 solid tumor	1700 folds higher cellular uptake of miR-34a compared to blank NPs Superior in vivo cytotoxicity	[184]
	HA	Polymer–drug conjugate	Rapamycin	In vitro/MDA-MB-468 cells In vivo/Balb/c mice bearing CD44-positive 4T1.2neu breast cancer	-3.2-folds higher cellular uptake than free drug -Significantly higher in vitro cell viability reduction -Improved animal survival and suppressed tumor growth in vivo -2.96-fold greater area under the curve (AUC) than that of the free drug -8.82-fold slower total body clearance	[111]
	HA	PLGA NPs	Docetaxel	In vitro/MDA-MB-231 cancer cell line In vivo/Balb/c nude mice bearing MDA-MB-231 breast cancer	Significantly higher cellular uptake compared to nontargeted NPs Significantly enhanced tumor targeting and antitumor activity compared to nontargeted NPs	[112]
	HA, oligosaccharide (oHA)	Lipid NPs	Paclitaxel	In vitro/BT549, MDA-MB-231, MDA-MB-468, and T47D human breast cancer cell line In vivo/female nude mice bearing MDA-MB-231 breast tumor	Significantly improved antitumor activity	[113]
	HA	PAMAM dendrimers	DOX and major vault protein (MVP) targeted small interfering RNA (MVP-siRNA)	MCF-7/ADR breast cancer cells, Female Balb/c nude mice	-Significantly higher AUC and MRT, -Significantly enhanced cellular uptake in vitro ~4-fold reduction in IC_50_ value, improved gene silencing effect as well as enhanced stability and efficient intracellular delivery of siRNA	[185]
LHRH	LHRH-peptide	Dextran NPs	Cisplatin	In vitro/4T1 breast cancer cell line In vivo/Balb/c mice bearing 4T1 tumor	Significantly higher cellular uptake Significantly higher in vitro and in vivo cytotoxicity and low systemic toxicity	[119]
	LHRH-peptide	Human serum albumin NPs	Methotrexate	In vivo/Female Balb/c mice bearing 4T1 breast cancer	7-fold stronger antitumor efficacy 2-fold increase in the life span of mice	[120]
Transferrin	Tf	MSNs	DOX	HT-29 and MCF-7 cells	Receptor-mediated internalization of the drug in cancer cells Drug release is triggered by high GSH concentration in tumor cells.	[124]
	Tf	Polymeric NPs	Benzoporphyrin derivative monoacid (BPD)	TNBC cell line MDA-MB-231 breast epithelial cell line MCF-12A	-Specificity of the targeted NPs for TNBC cells -Highest photo triggered cytotoxicity in TNBC cells	[186]
	Tf	NPs	Curcumin and DOX	MCF-7 breast cancer cells and mice bearing MCF-7 cells	-In vitro cell viability assay exhibited higher cytotoxicity -Inhibition of viability and proliferation of the cancer cell lines with lower IC_50_ value. -Higher drug concentrations in the tumor tissue owing to EPR effect.	[125]
	Tf	Polymeric NPs	Nutlin-3a	MCF-7 breast cancer cells	22-times and 3-fold higher uptake than native nutlin-3a and unconjugated NPs, superior antiproliferative activity	[126]
	Tf	Lipid coated PLGA NPs	7α-(4′-amino) phenylthio-1,4-androstadiene-3,17-dione (7α-APTADD)	In vitro/SK-BR-3 breast cancer cells	Significantly higher cellular uptake 2.8-folds reduction in IC_50_ compared to the nontargeted NPs	[127]
	Tf	SLNs	Curcumin	In vitro/MCF-7 breast cancer cell line	2- and 5-fold higher cellular uptake compared to nontargeted NPs and free drug solution 1.5- and 3-fold higher cytotoxicity compared to nontargeted NPs and free drug solution	[128]
Integrin	Leukocytes	Biomimetic nanovesicles (leukosomes)	DOX	4T1 and B16 cancer cell lines	Significantly higher tumor accumulation for leukosomes More potent anticancer activity in terms of reduction of tumor volume and prolonged survival	[133]
	RGD motif (Arg-Gly-Asp)	Lipid polymer hybrid NPs	Norcantharidin	Human TNBC cell lines MDA-MB-231, LM2, and SUM159 cells Nude mouse orthotopic mammary TNBC tumor	Specific β-catenin attenuation Significantly enhanced accumulation and longer retention time	[187]
	RGD motif (Arg-Gly-Asp)	Polymeric dendritic NPs	DOX	Mouse mammary breast tumor cell line (4T1) HUVEC cells Female BALB/C mice	-Accumulation of drug around the leaky blood vessels, -Shrinkable property of formulation is beneficial for penetration and retention -In vivo, RGD-DOX-DGL-NPs showed a remarkable tumor growth inhibition effect	[188]
	Fibronectin-mimetic peptide	PEGylated liposomes	DOX	In vitro/MDA-MB-231 breast cancer cells	Enhanced binding efficacy Enhanced cytotoxicity	[74]
	cyclic pentapeptide c(RGDfK)	PLGA-PEG NPs	Cisplatin	In vitro/MCF-7, MCF-7MFP1, DU145, DU145LN2, PC3, PC3MLN4 cell lines In vivo/female nude mice xenograft bearing MCF-7MFP1 breast cancer cell	Significantly higher cellular uptake Significantly higher cytotoxicity in vitro and in vivo	[75]
Vasoactive Intestinal Peptide (VIP) receptor	VIP	Sterically stabilized phospholipid nanomicelles	Curcumin	Breast cancer stem cells MCF-7 human breast cancer cell line	Significantly improved IC_50_ 20% inhibition of tumorsphere formation Significantly enhanced anticancer activity	[139]
	VIP	Nanomicelles	Paclitaxel	MCF-7 breast cancer cells/Virgin female Sprague-Dawley rats	Significantly higher cellular uptake, 2-fold improvements in ED_50_ value compared to free paclitaxel, improved in vivo anticancer efficacy	[140]
	VIP	Nanomicelles	Paclitaxel	MCF-7 breast cancer cells, BC19/3 breast cancer cells	Significantly enhanced cytotoxicity in vitro	[141]
	VIP	Nanomicelles	17-Allylamino-17-demethoxy geldanamycin	MCF-7 breast cancer cells	Significantly enhanced cytotoxicity in vitro	[142]
Heparin-binding epidermal growth factor (HB-EGF) receptor	Fab’ antibody 8 against heparin-binding EGF growth factor	Lipid NPs	Si-RNA	MDA-MB-231 human TNBC cells	In vivo studies showed long-term blood circulation and accumulation in the tumor tissue Suppression of PLK1 protein expression and tumor growth	[189]
	Antihuman heparin-binding epidermal growth factor (HB-EGF) monoclonal antibody	Immunoliposomes	DOX	Vero-H cells, MDA-MB-231 human breast cancer cells/Balb/c nude female mice	Significantly higher cellular uptake in vitro, strong suppression, and regression of breast tumor	[146]
N-acetyl-D-glucosamine (NAG) receptor	NAG	Polymeric NPs	DOX	MCF-7 breast cancer cell line	Significantly higher cellular uptake and targeting ability, higher antitumor activity	[143]
EphA2 receptor	Homing peptide with a sequence of YSAYPDSVPMMSK	MSNs	DOX	MCF-7 cell lines	-Increased specificity and cytotoxicity of DOX in MCF-7/MDR1 cells in vitro and in vivo -Reduced toxicity and enhanced therapeutic efficacy	[190]
	Homing peptide with a sequence of YSAYPDSVPMMSK	Liposomes	DOX	MDA-MB-231, HUVEC cells Balb/c nude mice	-Significantly higher cellular uptake In vitro, stronger cytotoxicity in vitro and In vivo, low systemic and cardiac toxicity	[144]
Alpha7 nicotinic acetylcholine receptor (α7 nAChR)	α-conotoxin ImI	Micelles	Paclitaxel	A549 breast cancer cells, MCF-7 breast cancer cells/female Balb/c nude mice	-Significantly higher cellular uptake in vitro, -Significantly higher cytotoxicity in vivo, -Low systemic toxicity	[145]
Biotin Receptor	Double branched Biotin	Liposomes	Paclitaxel	MCF-7 cells (Human breast cancer cell line), 4T1 cells (Mouse breast cancer cell line) B16 cells (Mouse skin melanoma cell line) Female Balb/c mice	-Excellent targeting ability to breast cancer. -The relative uptake efficiency (RE) and concentration efficiency (CE) of (Bio2-Chol) Lip were respectively enhanced by 5.61- and 5.06-fold compared to that of naked paclitaxel.	[191]
	Biotin	PEG-b-poly (ε-caprolactone) NPs	DOX and Quercetin	MCF-7/ADR cell lines	Facilitates the cellular drug uptake and reduces the drug efflux rate Inhibition of both P-gp activity and expression	[192]
	Biotin	DNA conjugated gold nanorods (GNR)	DOX	MCF-7/ADR cell lines	-Increased cell uptake and significantly reduced drug efflux, -About 67-fold increased potency than free drug	[193]
	Biotin	liposomes	DOX and Quercetin	MCF-7/ADR cell lines	-Higher antitumor activity -Decreased cardiotoxicity of DOX -Downregulation of P-gp expression In vivo.	[194]
	Biotin	Human serum albumin NPs	Methotrexate	Balb/c mice bearing 4T1 breast carcinoma	-Significantly stronger anticancer activity and lower toxic effect, -Increased survival and life span of tumor-bearing mice, slight body weight loss	[147]
KDR receptor	K237-peptide	Hybrid chitosan/poly(N-isopropylacrylamide) NPs	Paclitaxel	MDA-MB-231 human breast cancer cells	-MTT assays showed that the K237-conjugated NPs could more effectively inhibit breast cancer cell growth -Enhanced efficacy in preventing cell proliferation	[195]
	K237- (HTMYYHHYQHHL) peptide	Polymeric NPs	Paclitaxel	HUVEC cells, MDA-MB-231 cells, Female Balb/c nude mice	-Significantly higher cellular uptake, -Significantly stronger antitumor efficacy in vitro and In vivo	[148]
-	Cyclic Arg-Gly-Asp-d-Tyr-Lys [c(RGDyK)]	Polymeric micelles	DOX	BCap-37 cells and Bcap37 cells bearing female BALB/c mice	Developed micelles system prolonged drug half-life in bloodstream, improved therapeutic efficiency, and decreased cardiac toxicity and biotoxicity compared to free drug.	[196]

## Data Availability

Not applicable.

## List of Abbreviations

ABC	ATP binding cassette
BCRP	Breast cancer resistance protein
Cur	Curcumin
DOX	Doxorubicin
EGFR	Epidermal growth factor receptor
ER	Estrogen receptor
FDA	Food and drug administration
FR	Folate receptor
HA	Hyaluronic acid
HER2	Human epidermal growth factor receptor 2
HR	Hyaluronan receptor
IR	Integrin receptor
LHRH	Luteinizing hormone-releasing hormone receptor
MDR	Multidrug resistance
miRNA	microRNA
MNPs	Magnetite nanoparticles
MRP1	MDR associated protein
NAG	N-acetyl-D-glucosamine
NLC	Nanostructured lipid carriers
P-gp	P-glycoprotein
PTX	Paclitaxel
siRNA	Small interfering RNA
SLN	Solid lipid nanoparticles
SNEDDS	Self-nano emulsifying drug delivery system
TfR	Transferrin receptor
TNBC	Triple-negative breast cancer
TPGS	D-α-Tocopheryl polyethylene glycol 1000 succinate
VIP	Vasoactive intestinal peptide receptor
WHO	World Health Organization
